# Immediate chromatin immunoprecipitation and on-bead quantitative PCR analysis: a versatile and rapid ChIP procedure

**DOI:** 10.1093/nar/gku1347

**Published:** 2014-12-24

**Authors:** Kayla M. Harmeyer, Paul F. South, Brett Bishop, Joe Ogas, Scott D. Briggs

**Affiliations:** Department of Biochemistry and Purdue University Center for Cancer Research, Purdue University, West Lafayette, IN 47907, USA

## Abstract

Genome-wide chromatin immunoprecipitation (ChIP) studies have brought significant insight into the genomic localization of chromatin-associated proteins and histone modifications. The large amount of data generated by these analyses, however, require approaches that enable rapid validation and analysis of biological relevance. Furthermore, there are still protein and modification targets that are difficult to detect using standard ChIP methods. To address these issues, we developed an immediate chromatin immunoprecipitation procedure which we call ZipChip. ZipChip significantly reduces the time and increases sensitivity allowing for rapid screening of multiple loci. Here we describe how ZipChIP enables detection of histone modifications (H3K4 mono- and trimethylation) and two yeast histone demethylases, Jhd2 and Rph1, which were previously difficult to detect using standard methods. Furthermore, we demonstrate the versatility of ZipChIP by analyzing the enrichment of the histone deacetylase Sir2 at heterochromatin in yeast and enrichment of the chromatin remodeler, PICKLE, at euchromatin in *Arabidopsis thaliana*.

## INTRODUCTION

Characterizing the dynamics of the chromatin structure and associated proteins is fundamentally important in the understanding of cellular growth and differentiation. Chromatin is the protein–DNA complex that packages DNA in the nucleus of eukaryotic cells. The basic unit of chromatin is the nucleosome, which is composed of 146 base pairs of DNA wrapped around an octamer of histone proteins (1,2). Changing how the DNA is packaged in the nucleus affects many DNA-templated processes, including gene transcription and DNA repair and replication. As a result, detailed analysis of how chromatin features change in time and space is critical in understanding how these processes are regulated (2–4). Changes in DNA packaging can be modulated by a variety of chromatin-associated proteins, such as transcription factors and remodelers, and also by post-translation modifications (PTMs) on histones (1,4–6).

The development of chromatin immunoprecipitation (ChIP), ChIP-chip and ChIP-seq has led to a significant increase in the amount of information gained about the nature of chromatin in respect to the localization of histone variants and of chromatin-associated proteins in the genome (3,7). Furthermore, these methods have revealed the enrichment pattern of PTMs such as methylation, acetylation and phosphorylation across the genome, which has shed new light on how these epigenetic modifications impact cellular processes (8). ChIP has also helped to elucidate how these modifications act as docking or recruitment sites for transcription factors, chromatin remodelers and even other chromatin modifiers, thereby contributing mechanistic insight into regulation of chromatin dynamics (1,2,4,6,7).

The successful application of ChIP-chip and ChIP-seq has resulted in a significant increase in the amount of genome-wide information that has been generated and collected for a variety of organisms. With the facile ability to generate vast amounts of genome-wide ChIP data, it has become increasingly important to rapidly validate these data. Unfortunately, standard ChIP methods are time consuming, expensive, quite laborious and are subject to high rates of experimental error due to the large number of steps involved. Standard ChIP analysis typically takes 3–4 days, from growing cultures to data analysis (9). To reduce the time of ChIP analysis, other groups have developed ‘Fast’ ChIP protocols that can significantly reduce the time to complete an experiment (10–12). One reported fast ChIP protocol showed that incubation of antibody with chromatin in an ultrasonic bath significantly reduces incubation time (10,11). This protocol also included treating the precipitated chromatin at 100°C with chelex-100 resin to extract the DNA from proteins (10,11). This approach can be used in both standard polymerase chain reaction (PCR) and in quantitative real-time PCR (qRT-PCR), but the 15-min incubation time in an ultrasonic bath may not be sufficient for some antibody–epitope interactions, which would significantly decrease the sensitivity of the ChIP procedure. In addition, the use of chelex-100 resin not only leads to additional time and cost, but contamination of a PCR reaction with chelex-100 resin prevents DNA amplification (13). Another ChIP procedure demonstrated that after immunoprecipitation, the chromatin does not need to be eluted from the beads and these beads can be directly used for PCR (12). This protocol can be used for standard PCR, but it was thought not to be compatible with qRT-PCR due to background fluorescence caused by the beads. Furthermore, even with these advancements in performing ChIP, there are still protein factors that have been difficult or nearly impossible to detect (14–16). Because of these issues with the current ChIP protocols, there is a need for a ChIP protocol that not only decreases the amount of time and cost, but also increases the sensitivity allowing for the study of the proteins and PTMs that have been traditionally difficult to detect. Here we report a new ChIP procedure, which we call ZipChIP.

Using ZipChIP one can take soluble chromatin to samples ready for qRT-PCR data analysis in approximately 2.5 h without the use of an ultrasonic bath or chelex-100 resin. Furthermore, we have optimized the procedure to allow the use of magnetic beads in an on-bead PCR reaction using qRT-PCR to improve the sensitivity of the method. We show that the ZipChIP method can successfully detect the highly abundant PTM histone H3 lysine 4 (H3K4) trimethylation in a similar enrichment pattern as produced by the standard ChIP protocol. Next, we compared ZipChIP versus standard ChIP to detect two yeast histone demethylases, Jhd2 and Rph1, to demonstrate the increased sensitivity of the ZipChIP protocol. Furthermore, we used ZipChIP to analyze the enrichment of the histone deacetylase Sir2 at a subtelomere gene using a Sir2-specific antibody. We also scanned the promoter and open-reading frame (ORF) of four actively transcribed genes for an enrichment pattern of H3K4 monomethylation and show that three of the six active genes had a 3′-ORF enrichment pattern. Taken together, ZipChIP analysis indicates that there are at least two patterns of H3K4 monomethylation on actively transcribed genes. We also analyzed the H3K4 monomethylation enrichment pattern in yeast strains with gene deletions that lead to a loss of H2B monoubiquitination. These data demonstrate the ability of ZipChIP to screen through multiple genes and yeast strains to shed light on a biological question of how a PTM can affect the enrichment pattern of another PTM across a gene's promoter and ORF. Finally, we show the versatility of ZipChIP on the more complex genome of *Arabidopsis thaliana*. PICKLE (PKL), a CHD subfamily II ATP-dependent chromatin remodeling factor, is correlated with the deposition of H3K27 methylation, a mark commonly associated with transcriptional repression. However, PKL also associates with the actively transcribed gene *ACT7* (17–19). We show that this association with *ACT7* does not correlate with H3K4 trimethylation, a PTM associated with active transcription. These studies show that the ZipChIP procedure serves as a powerful tool to answer biological questions in a timely and cost-effective manner with increased sensitivity.

## MATERIALS AND METHODS

### Yeast strains

All yeast strains used in this study are described in Supplementary Table S1. All strains were grown overnight to saturation in YPD media. They were then back diluted to OD_600_ 0.1–0.2 in 100-ml YPD and grown at 30°C to mid-log phase (OD_600_ 0.6–0.8).

### Formaldehyde cross-linking and generating soluble chromatin from *Saccharomyces cerevisiae*

The generation of soluble chromatin was described previously (9). The cultures were cross-linked with 1% formaldehyde for 15 min at room temperature, mixing occasionally. The cells were then collected with centrifugation and washed. The cells were again collected with centrifugation, transferred to a microcentrifuge tube and collected with centrifugation. The cell pellet was flash-frozen in liquid nitrogen and stored at −80°C. The cell pellets were resuspended in 400 μl sFA-140 lysis buffer [sFA-140 refers to the FA-140 buffer used for *Saccharomyces cerevisiae*; 50 mM HEPES-KOH, pH 7.5; 140 mM NaCl; 1 mM ethylenediaminetetraacetic acid (EDTA); 0.1% Triton X-100 and 0.1% sodium deoxycholate]. The cells were lysed by bead-beating with glass beads (BioSpec 11079105). The lysate was separated from the beads and sonicated (Misonix sonicator: Power 6, 10 s, six times with 10-s rest) to shear the chromatin. Directly after sonication, the lysate was placed on dry ice for 10 s. The lysates were centrifuged for 5 min at 4°C at top speed and then for 10 min at 4°C at top speed to separate soluble and insoluble chromatin (∼400 μl). Twenty five microliters (6.25%) of soluble chromatin was removed and processed for DNA input using a PCR purification kit (Qiagen 28106). The remaining soluble chromatin can be immediately used for the immunoprecipitation or it can be stored at −20°C.

### *S. cerevisiae* ZipChIP

Individual antibodies (1 μl H3, Abcam ab1791; 1 μl H3K4me3, Millipore 07-473; 1 μl H3K4me1, Active Motif 39297; 1 μl M2 FLAG, Sigma F1804; 10 μl Sir2 (y-80), Santa Cruz sc-25753) were conjugated to 10 μl Protein-G magnetic beads (Dynabeads, Life Technologies 10004D) for about 30 min. To determine the amount of antibody used for immunoprecipitation, each antibody was titrated to assess the best signal-to-noise ratio. The antibody-bound beads were then rotated with soluble lysate [100 μl for H3; 25 μl for H3K4me3; 300 μl for H3K4me1; 200 μl for FLAG; 500 μl for Sir2; for H3 and H3K4me3, the soluble lysate was brought up to 200 μl total volume with 1xPBS(-K)] for 2 h at 4°C. The beads were then washed at room temperature for 5 min each with sFA-140 wash buffer (50 mM HEPES-KOH, pH 7.5; 140 mM NaCl; 1 mM EDTA; 0.1% Triton X-100), sFA-500 wash buffer (50 mM HEPES-KOH, pH 7.5; 500 mM NaCl; 1 mM EDTA; 0.1% Triton X-100) and with 1xPBS(-K), pH 7.0 (39 mM NaH_2_PO_4_·H_2_O; 32.3 mM Na_2_HPO_4_·7 H_2_O; 154 mM NaCl). Fifty microliters of water was used to resuspend the beads.

### *S. cerevisiae* standard ChIP

The standard ChIP protocol was performed as previously described with slight modifications (9,20,21). Soluble lysate (100 μl for H3; 25 μl for H3K4me3; 200 μl for FLAG; for H3 and H3K4me3, the soluble lysates were brought up to 200 μl total volume with sFA-140 lysis buffer) was rotated with 1 μl antibody (H3, Abcam ab1791; H3K4me3, Millipore 07-473; M2 FLAG, Sigma F1804) overnight at 4°C with protease inhibitors (1 μg/ml leupeptin, aprotinin and pepstatin A, 1 mM phenylmethylsulfonyl fluoride (PMSF). Similar to the antibodies, to determine the amount of lysate used per immunoprecipitation, different volumes of lysate were tested to determine the amount that would result in the best signal-to-noise ratio. Twelve microliters of Sepharose Protein-G beads (GE Healthcare Life Sciences 17-0618-02) was added to each lysate and incubated for 1 h at 4°C. The beads were then washed at room temperature for 10 min with sFA-140 wash buffer, sFA-500 wash buffer and with LiCl/NP40 (10 mM Tris–HCl, pH 8.0; 250 mM LiCl; 0.5% NP-40; 0.5% sodium deoxycholate; 1 mM EDTA). The bound protein and DNA was eluted from the beads using 200 μl elution buffer [1% sodium dodecyl sulphate (SDS); 0.1 M NaHCO_3_] by briefly vortexing the beads followed by 15-min rotation at room temperature. This was repeated, and the two elution fractions were combined. After combining the two elutions, 16 μl 5 M NaCl was added to each sample. For DNA input, 10 μl of soluble chromatin was used. Both the elutions and the inputs were reverse cross-linked overnight at 65°C. The DNA was then ethanol precipitated and dried. One hundred eighty microliters of Tris-EDTA (TE), pH 8.0 (10 mM Tris; 1 mM EDTA) was used to resuspend the DNA followed by RNase treatment (20 μg; Sigma R6513) for 30 min at 37°C. Twenty microliters of proteinase K buffer (0.1 M Tris–HCl, pH 7.8; 0.05 M EDTA; 5% SDS) was added followed by proteinase K treatment (20 μg; Sigma P2308) for 1 h at 42°C. The DNA was extracted with two phenol/chloroform/IAA washes. The DNA was again ethanol precipitated, dried and resuspended in 50 μl of water.

### *S. cerevisiae* real-time PCR and statistical analysis

qRT-PCR was performed using the StepOnePlus Real-Time PCR System (Life Technologies). To ensure equal amounts of DNA loaded, the samples were regularly vortexed to ensure suspension of the beads. Also to be noted, low levels (0.1% Triton X-100) must be used in the sFA-140 wash buffer and sFA-500 wash buffer to reduce background fluorescence caused by detergents when performing an on-bead PCR reaction. One microliter of the on-bead mixture and standard ChIP DNA were used for each of the three technical repeats for each of the three biological repeats. The DNA was amplified using TaqMan Master Mix (Life Technologies 4369510) and PrimeTime probe sets (Integrated DNA Technologies) described in Supplementary Table S2. The data were analyzed using the ΔΔCt method. The mean relative fold changes, SEM and statistical significance values are found in Supplementary Tables S4–S13.

### *A. thaliana* material and growth conditions

Seeds used in these studies were obtained from wild-type (WT) or transgenic plants grown in parallel in an AR75 incubator (Percival Scientific) under 24 h of illumination. Seeds were allowed to dry for at least a month on the plant prior to collection. No other treatment was applied (i.e. stratification) prior to use of the seeds. Plants were incubated on synthetic media (22) and grown in a CU36L5 incubator (Percival Scientific) under 24 h of illumination. Whole plants were collected 14 days post imbibition for ChIP analysis.

### Isolating nuclei and generating soluble chromatin from *A. thaliana*

The nuclei extraction and generation of soluble chromatin from *A. thaliana* were performed as previously described (17). Tissue was immersed in cross-linking solution (0.002% Silwet; 10 mM Tris–HCl, pH8.0; 0.44 M sucrose; 1% formaldehyde) under house vacuum. After 10 min, 1/15 volume of 2 M glycine was added, and the vacuum was reapplied for another 5 min. The tissues were washed and flash-frozen in liquid nitrogen. The tissue was ground into a fine powder and resuspended in 10 ml of cold Extraction Buffer 1 (0.4 M sucrose; 10 mM Tris–HCl, pH 8.0; 10 mM MgCl_2_) with protease inhibitors (1x protease inhibitor cocktail; Sigma P9599). The following steps were performed at 4°C or on ice unless otherwise indicated. The lysate was filtered through one layer of cheesecloth (VWR 21910-107). After filtering, the cheesecloth was rinsed with another 10 ml of cold Extraction Buffer 1. All 20 ml of Extraction Buffer 1 containing tissue samples were then filtered through one layer of Miracloth (Calbiochem 475855) and centrifuged at 3000x g for 20 min. The pellet was resuspended in 1.5 ml of Extraction Buffer 2 (0.25 M sucrose; 10 mM Tris–HCl, pH 8.0; 10 mM MgCl_2_; 1% Triton X-100) with protease inhibitors and centrifuged at 2000x g for 10 min. Each pellet was resuspended in another 1 ml of Extraction Buffer 2 with protease inhibitors and centrifuged at 2000x g. Each pellet was then resuspended in 600 μl of Extraction Buffer 3 (1.7 M sucrose; 10 mM Tris-HCl, pH 8.0; 2 mM MgCl_2_; 0.15% Triton X-100) with protease inhibitors, layered on top of another 600 μl of Extraction Buffer 3 and centrifuged (16 000x g, 1 h). Pellets from each tube were resuspended in 750 μl of HEPES buffer (50 mM HEPES-KOH, pH 7.5; 140 mM NaCl; 1% Triton X-100; 0.1% Na deoxycholate; 1 mM EDTA) with protease inhibitors and the chromatin was sheared into ∼250–750 bp fragments by sonication (Misonix sonicator: Power 6, 10 s, six times with 20-s rest). The tubes were then centrifuged (16 000x g, 10 min), and the supernatant was transferred to a new tube.

### *A. thaliana* ZipChIP protocol

One microliter of antibody (anti-H3, Abcam ab1791; anti-H3K4me3, Millipore 07-473), or no antibody was conjugated to 10 μl Protein-G magnetic beads (Dynabeads, Life Technologies 10004D; equilibrated with PBS-T, 50% slurry) for about 30 min. Before performing the immunoprecipitation, the soluble lysate was precleared by rocking with 5 μl of Protein-G magnetic beads (Dynabeads, Life Technologies 10004D; equilibrated with HEPES buffer, 50% slurry) at 4°C for 30 min. Two hundred microliters of the precleared supernatant was added to the beads conjugated with the respective antibodies and incubated for 2 h. The beads were washed sequentially with 1 ml of each of the following: aFA-140 wash buffer (aFA-140 buffer refers to the FA-140 buffer used for *A. thaliana*; 50 mM HEPES-KOH, pH 7.5; 140 mM NaCl; 1% Triton X-100; 1mM EDTA), aFA-500 wash buffer (as HEPES buffer, but with 500 mM NaCl and 0.1% Na deoxycholate) and 1xPBS-T. For the *A. thaliana* ZipChIP, on-bead analysis was not used due to the use of SYBR Green Universal Master Mix for detection of PCR amplification. The immunocomplexes were eluted by heating at 100°C for 10 min. The samples were cooled to room temperature. The tubes were then spun at 12 000x g for 1 min and the supernatant removed to a new tube.

### *A. thaliana* real-time PCR and statistical analysis

qRT-PCR was performed using the StepOnePlus Real-Time PCR System (Life Technologies). Four microliters of DNA was used for each of the three technical repeats for each of the three biological repeats. The DNA was amplified using SYBR Green Universal Master Mix (Life Technologies) and the primers described in Supplementary Table S3. The data were analyzed using the ΔΔCt method for PKL-cMYC and the ΔCt method for H3K4me3. The mean relative fold changes or ΔCt values, SEM and statistical significance values are found in Supplementary Tables S14 and S15.

## RESULTS

### Verification of ZipChIP through analysis of the pattern of H3K4 trimethylation across actively transcribed genes

**Figure 1.**
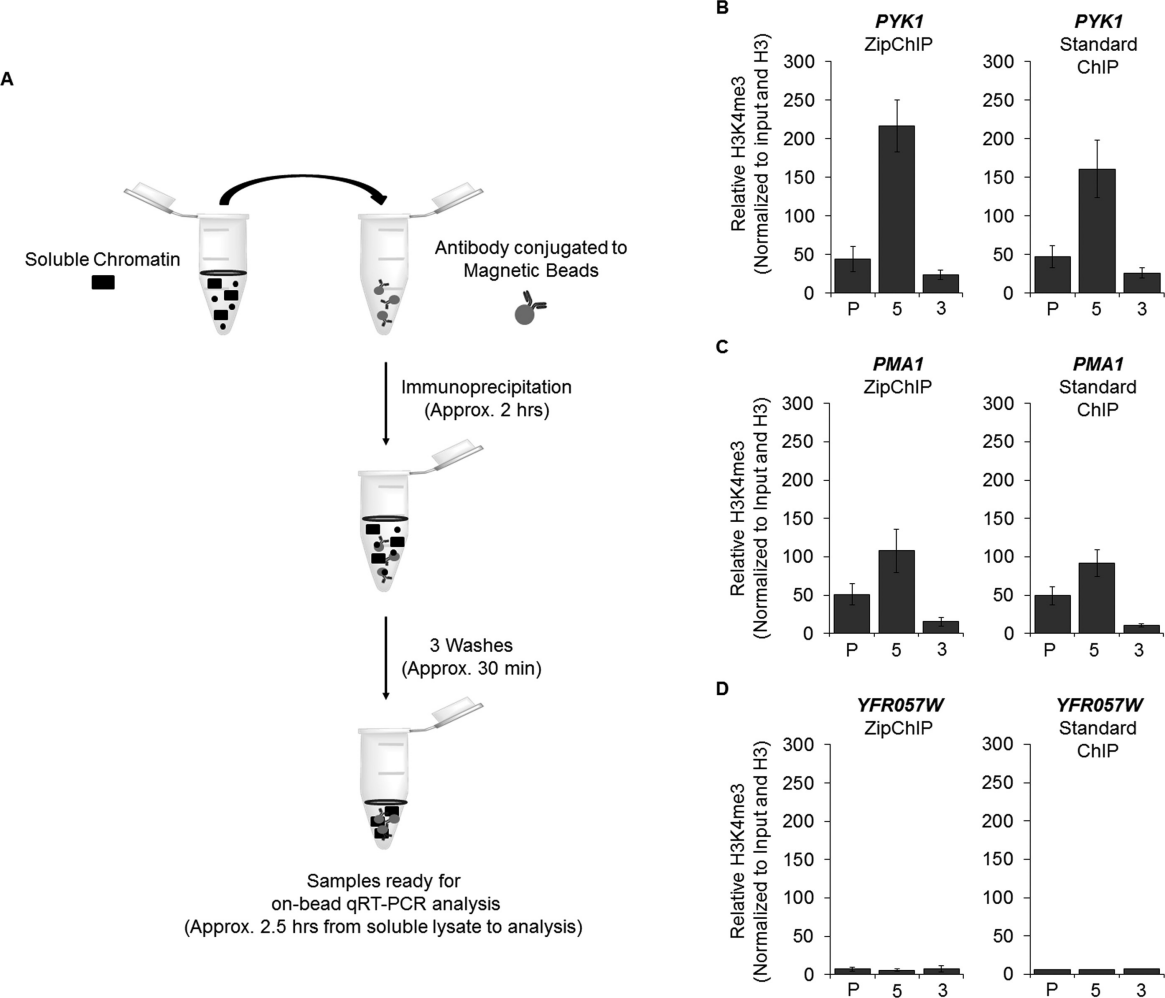
ZipChIP reduces the time from soluble chromatin to analysis to approximately 2.5 h. (**A**) Flow diagram depicting the steps of ZipChIP starting at soluble chromatin to samples ready for qRT-PCR analysis. (**B**–**D**) PrimeTime qPCR probes were targeted toward the promoter (P), 5′-ORF (5) and 3′-ORF (3) of two actively transcribed genes *PYK1* and *PMA1*, as well as a subtelomere gene *YFR057W* comparing ZipChIP and standard ChIP methods. ChIP analysis was performed on BY4741 WT and *set1Δ* strains using antibodies specific for H3K4me3 and histone H3. Input and histone H3 were used for normalization. ChIP analysis is relative to the H3K4me3 signal for the *set1Δ* strain. Three biological replicates with three technical repeats were used for all ZipChIP and standard ChIP analyses. The error bars represent the standard error of the mean.

Many studies have characterized a specific pattern of H3K4 methylation across the ORF of actively transcribed genes, with H3K4 trimethylation (H3K4me3) being enriched at the 5′-ORF and transcriptional start site, H3K4 dimethylation (H3K4me2) localized in the middle of the gene and H3K4 monomethylation (H3K4me1) enriched at the 3′-ORF (8,23). To establish that ZipChIP analysis is a reliable and robust method, ZipChIP was compared to a standard ‘long’ ChIP method (9,20,21) using an H3K4me3-specific antibody. The general scheme of the ZipChIP protocol is depicted in Figure 1A. Yeast cells were grown to log phase and cross-linked with formaldehyde. After cell lysis and sonication, chromatin was purified. For the DNA input control, 6.25% of soluble chromatin was removed and processed with a standard PCR clean up kit (Qiagen). During the chromatin preparation, the H3K4me3 antibody (Millipore) was conjugated to Protein-G magnetic beads (DynaBeads, Life Technologies) for 30 min. The soluble chromatin was then immunoprecipitated with the antibody-conjugated magnetic beads for 2 h. After immunoprecipitation, the beads were washed three times for 5 min each and resuspended in water. The H3K4me3 ZipChIP sample was then analyzed using qRT-PCR at the promoter, 5′-ORF and 3′-ORF of two constitutively active genes, *PYK1* and *PMA1* (Figure 1B and C). As a negative control, H3K4me3 ZipChIP was performed in a yeast strain lacking the only known H3K4 histone methyltransferase, Set1. In the *set1Δ* strain, H3K4 mono-, di- and trimethylation are abolished (24). A standard ChIP method was also used to analyze the H3K4me3 pattern across these two genes in both a WT strain and a *set1Δ* strain (Figure 1B and C and Supplementary Figure S1). Both methods showed the characteristic peak of H3K4me3 at the 5′-ORF of both genes. Furthermore, the signal of the H3K4me3 detected was very similar between the two methods when normalizing to the H3K4 trimethylation signal for a *set1Δ* strain (Figure 1B and C), when normalizing to the H3K4 trimethylation signal at the promoter of the individual gene in the WT strain (Supplementary Figure S1D–S1E) and normalizing to the H3K4 trimethylation signal of the subtelomere gene *YFR057W* (Supplementary Figures S1G and S1H). This indicates that the ZipChIP method can be used to detect H3K4me3 in about 6 h after formaldehyde cross-linking compared to the 3 days using standard ChIP. As a control to show that the methylation detected at the two active genes *PMA1* and *PYK1* was specific, a silent subtelomere gene, *YFR057W*, was analyzed for H3K4me3. Both methods showed very low levels of H3K4me3 at *YFR057W* when compared to *PYK1* and *PMA1*, as expected due to the correlation between H3K4me3 and active transcription (Figure 1D and Supplementary Figure S1). To show H3K4me3 ZipChIP background levels in a *set1Δ* strain, the background signal for each gene was normalized to the promoter of each gene (Supplementary Figure S1A–S1C). Data generated in Figure 1 were also normalized to their respective gene promoter (Supplementary Figure S1D–S1F). In addition, *PYK1* and *PMA1* were also normalized with respect to the *YFR057W* promoter (Supplementary Figure S1G and S1H).

### Histone demethylases, Jhd2 and Rph1, associate with the promoter, 5′-ORF and 3-ORF of actively transcribed genes

**Figure 2.**
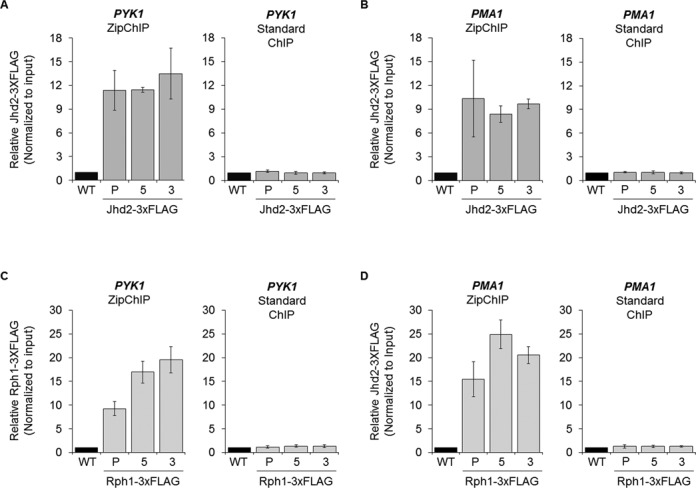
ZipChIP can detect a greater range of factors compared to standard ChIP. (**A** and **B**) PrimeTime qPCR probes were targeted toward the promoter (P), 5′-ORF (5) and 3′-ORF (3) of *PYK1* and *PMA1* comparing ZipChIP and standard ChIP methods that were performed on BY4741 WT strain and a *JHD2–*3xFLAG strain with the tag integrated at *JHD2*′s endogenous locus. (**C** and **D**) PrimeTime qPCR probes were targeted toward the promoter (P), 5′-ORF (5) and 3′-ORF (3) of *PYK1* and *PMA1* comparing ZipChIP and standard ChIP methods that were performed on BY4741 WT strain and a *RPH1–*3xFLAG strain with the tag integrated at *RPH1*′s endogenous locus. An α-FLAG antibody was used for immunoprecipitation of both Jhd2–3xFLAG and Rph1–3xFLAG for both ChIP methods. Input was used for normalization, and ChIP analysis is relative to a WT untagged control. Three biological replicates with three technical repeats were used for all ZipChIP and standard ChIP analyses. The error bars represent the standard error of the mean.

Studies have described protein factors that have been difficult or nearly impossible to detect using the standard ChIP protocol (14,16). For example, it has been reported that the yeast H3K4 histone demethylase Jhd2 cannot be analyzed using standard ChIP methods (15). However, using the ZipChIP protocol, we were able to readily detect Jhd2 and the H3K36 histone demethylase Rph1 interacting with the promoter, 5′-ORF and 3′-ORF of two actively transcribed genes, *PYK1* and *PMA1*, with a signal that was significantly over the untagged Jhd2 and untagged Rph1 background signal, respectively (Figure 2A–D). In contrast, a signal was not observed over background for either Jhd2 or Rph1 using the standard ChIP method (Figure 2A–D). To determine if the signal observed for Jhd2 at *PYK1* and *PMA1* is enriched over an intergenic region, Jhd2 was analyzed at an autonomously replicating sequence (*ARS504*). The levels of H3K4 demethylase Jhd2 were 3.3–5.3 fold higher at the actively transcribed genes compared to *ARS504* (Supplementary Figure S2A and S2B). These data show that Jhd2 is enriched at *PYK1* and *PMA1* compared to *ARS504*. Low levels of protein at *ARS504* were specific for Jhd2 as Rph1 was detected at this locus at similar levels to what was detected at *PYK1* and *PMA1* (Supplementary Figure S2C). The ability to analyze chromatin-associated proteins, which have been difficult to detect using standard ChIP, makes the ZipChIP method a significant and important tool for future work characterizing the association of these and other chromatin-associated proteins to specific loci.

To further establish that ZipChIP can show specific enrichment of proteins and to determine if ZipChIP can be used to determine enrichment of a chromatin-associated protein using a protein-specific antibody, levels of the histone deacetylase Sir2 were analyzed. This protein has been shown to localize at the silent genes including the subtelomere gene *YFR057W* (25). Therefore, a Sir2-specific antibody was used to assess Sir2 levels at *YFR057W* and at two actively transcribed genes *PYK1* and *PMA1* (Supplementary Figure S3A and S3B). Using ZipChIP, Sir2 enrichment was observed at *YFR057W* 3′-ORF and 3′-UTR (untranslated region) (Supplementary Figure S3A), and as expected low levels of Sir2 were observed at the 3′-ORFs of *PYK1* and *PMA1* (Supplementary Figure S3B). This further illustrates that ZipChIP is specific and works with antibodies that recognize epitope-tagged proteins or endogenous proteins.

### H3K4 monomethylation is enriched at the 3′-ORF for a subset of actively transcribed genes and this enrichment is lost in both *rad6Δ* and *bre1Δ* strains

**Figure 3.**
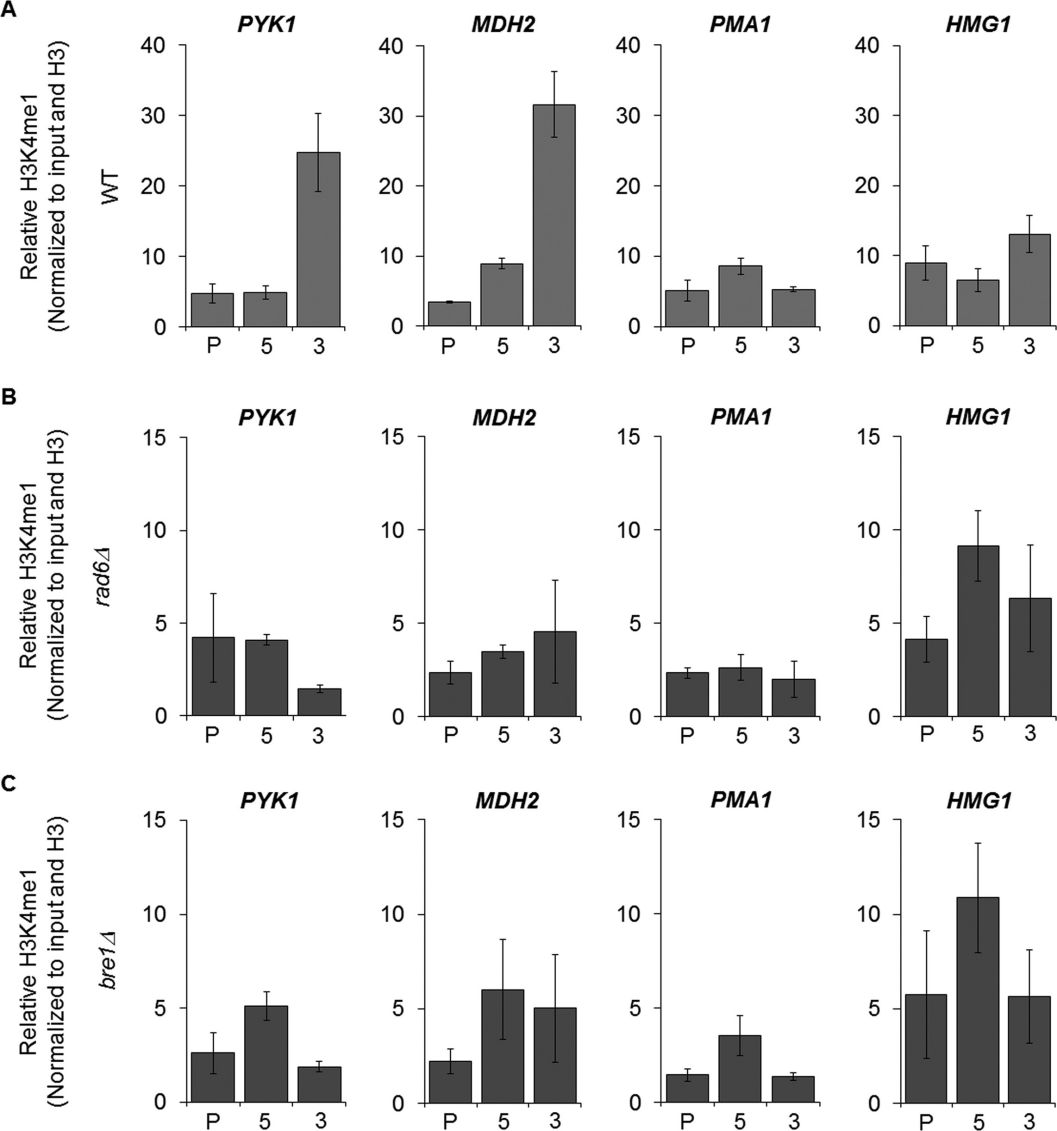
Two distinct patterns of H3K4 monomethylation were found using ZipChIP. (**A**–**C**) PrimeTime qPCR probes were targeted toward the promoter (P), 5′-ORF (5) and 3′-ORF (3) of *PYK1, MDH2, PMA1* and *HMG1*. (A–C) ZipChIP analysis was performed on BY4741 *set1Δ*, WT, *rad6Δ* and *bre1Δ* strains using antibodies specific for H3K4me1 and histone H3. Input and histone H3 were used for normalization. ZipChIP analysis is relative to the H3K4me1 signal for the *set1Δ* strain. Three biological replicates with three technical repeats were used for ZipChIP analysis. The error bars represent the standard error of the mean.

Although genome-wide approaches can provide valuable initial information, there are limitations with every technology. For example, reports of false positives due to sample bias and of low number of reads from certain targets necessitate validation and follow-up analysis of genome-wide localization data (26). As previously mentioned, H3K4me1 has been shown to be enriched at the 3′-ORF of actively transcribed genes (8,23). However, this particular histone modification has been challenging to study using standard ChIP in yeast, so its biological role is not fully understood. To determine if ZipChIP can be used to detect H3K4me1 in genomic regions, both WT and *set1Δ* strains were cross-linked and soluble chromatin was isolated from these strains. The Protein-G magnetic beads were conjugated with a commercially available H3K4me1 antibody (Active Motif) and used in the immunoprecipitation of soluble chromatin. Due to the lesser amount of this modification, the amount of soluble chromatin used in the immunoprecipitation was increased 1200% (300 μl) compared to that used for H3K4me3 ChIP (25 μl). After the ZipChIP method was completed, qRT-PCR analysis was used to analyze H3K4me1 levels at the promoter, 5′-ORF and 3′-ORF of six active genes that according to ChIP-chip data have H3K4me1 3′-ORF enrichment (*PYK1, PMA1, MDH2, HMG1, ERG11* and *HMG2*) (8). Interestingly, the H3K4me1 ZipChIP results differ from that of the ChIP-chip data. Relative to the background signal of a *set1Δ* strain, *PYK1, MDH2* and *ERG11* had an enrichment of H3K4me1 at the 3′-ORF, consistent with enrichment pattern previously reported (Figure 3A and Supplementary Figure S4A). However, though H3K4me1 was detected at the active genes *PMA1, HMG1* and *HMG2*, a 3′-ORF enrichment was not observed, differing from the previously reported ChIP-chip data (Figure 3A and Supplementary Figure S4A).

Due to the ability to detect significant levels of H3K4 monomethylation at actively transcribed genes using ZipChIP, we wanted to ask how other PTMs could affect the enrichment pattern of H3K4 monomethylation. The histone cross-talk between H3K4 methylation and H2B K123 monoubiquitination has been thoroughly studied, but still not completely understood. Multiple studies have shown that H2B monoubiquitination is necessary for the establishment of H3K4 di- and trimethylation (21,27–32). Global H3K4 di- and trimethylation were lost when *RAD6* or *BRE1*, the gene encoding the E2 ubiquitin-conjugating enzyme and the gene encoding the E3 ubiquitin ligase responsible for H2B monoubiquitination, respectively, was deleted (Supplementary Figure S4B). However, though global H3K4 monomethylation is reduced, it is still detectable through western blot analysis (21,28,29,32) (Supplementary Figure S4B). This suggests that though Rad6 and Bre1 affect the overall levels H3K4 monomethylation, some H3K4 monomethylation can be established even with their absence. How exactly Rad6 and Bre1 affect H3K4 monomethylation across a gene and if they play a role in the observed 3′ enrichment of H3K4 monomethylation is largely unknown. One study showed that in a *rad6Δ* an increase in H3K4 monomethylation was observed at the 5′-ORF of two actively transcribed genes, but also that H3 levels at the locus were higher in a *rad6Δ* compared to WT (29). This study also stated that no H3K4 monomethylation was observed at the 3′-ORF of the genes analyzed in a *rad6Δ* strain. Another study also looked at H3K4 monomethylation across a gene in both a *rad6Δ* and a *bre1Δ* strain, but reported that no change in signal was observed compared to WT (33).

To resolve these differences, ZipChIP was used to analyze the levels of H3K4 monomethylation in *rad6Δ* and *bre1Δ* strains across *PYK1* and *MDH2*, the two genes observed to have a 3′-ORF enrichment of monomethylation in a WT strain (Figure 3A). The ChIP analysis revealed that the 3′-ORF H3K4 monomethylation enrichment was lost at *PYK1* and *MDH2* in both *rad6Δ* and *bre1Δ* strains (Figure 3B and C). Interestingly, H3K4 monomethylation signal at the 3′-ORF of *PYK1* was significantly lower (*P* value < 0.05) compared to the 5′-ORF signal of *PYK1* in both *rad6Δ* and *bre1Δ* strains (Figure 3B and C). Furthermore, *PMA1* and *HMG1* were analyzed in a *rad6Δ* strain and *bre1Δ* strain, and as observed in the WT strain, levels of H3K4 monomethyation were similar across the promoter, 5′-ORF and 3′-ORF (Figure 3B and C). Taken together, ZipChIP was able to identify that there are two distinct patterns of H3K4 monomethylation and that H2B ubiquitination is necessary for H3K4 monomethylation enrichment at the 3′ ORF of some but not all genes.

### *A. thaliana* chromatin remodeler PKL localization does not correlate with H3K4 trimethylation enrichment at the active gene *ACT7*

**Figure 4.**
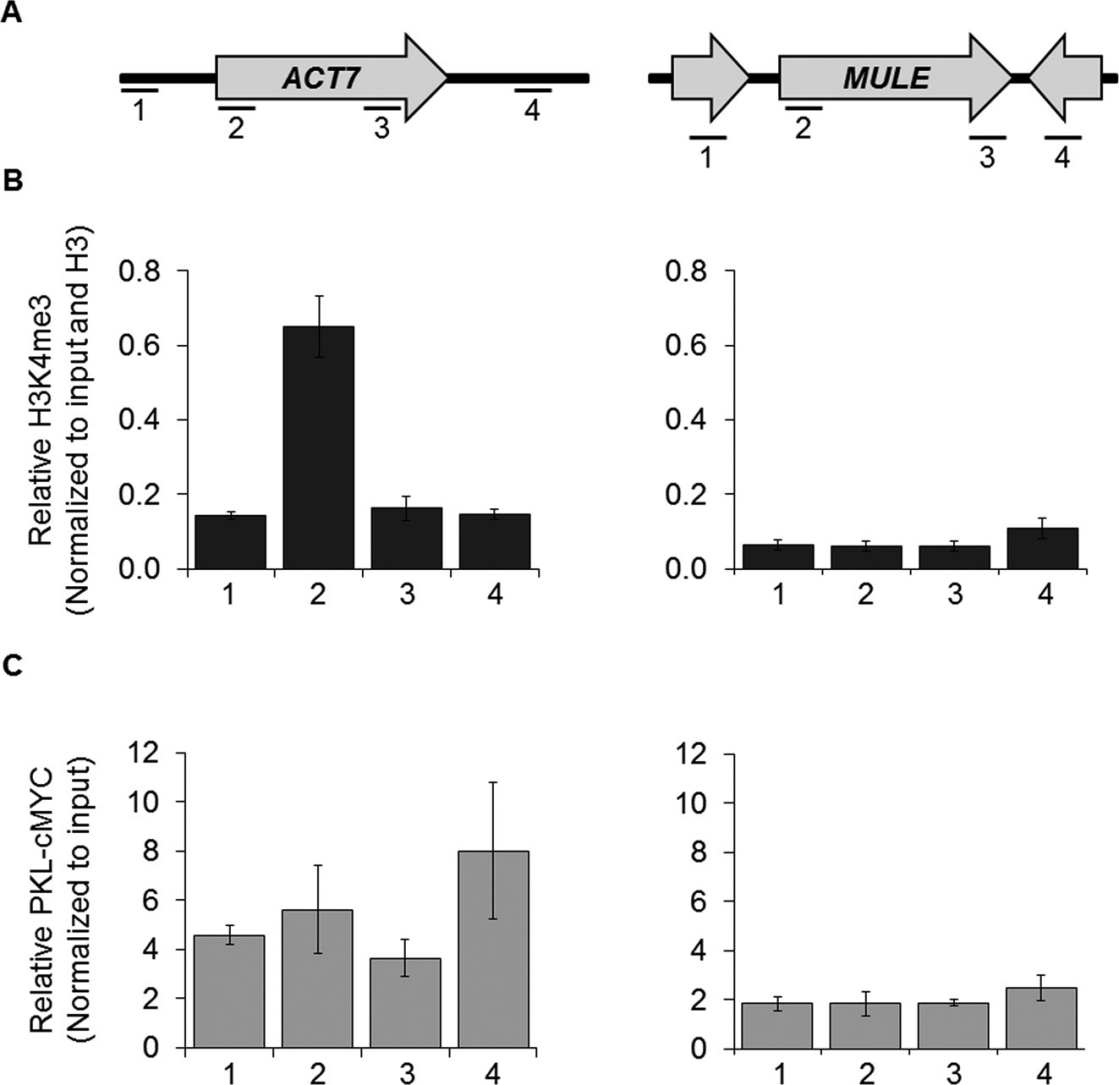
ZipChIP can be utilized in a more complex genome such as *A. thaliana*. (**A**) Schematic of the four different regions analyzed for H3K4me3 and PKL-cMYC using region-specific primers across the euchromatic gene *ACT7* and the heterochromatic gene *MULE*. (**B**) An antibody specific for H3K4me3 was used to detect methylation levels at both genes using ZipChIP. The H3K4me3 signal is normalized to input and histone H3. (**C**) ZipChIP analysis of PKL-cMYC was performed using a MYC-specific antibody, and the signal was normalized to input. Three biological replicates were used for ZipChIP analysis. The error bars represent the standard error of the mean.

We also wanted to test if ZipChIP could be used for more complex genomes. Here we show that the ZipChIP method can be applied to study the relationship between chromatin modifications and remodelers in *A. thaliana*. PICKLE (PKL) is an ATP-dependent remodeler that promotes H3K27me3, a mark associated with transcriptional repression (17). Interestingly, PKL not only associates with H3K27me3-enriched genes, but is also found at actively transcribed genes, such as the promoter of *ACT7* (17,34). The association of PKL with the promoter of *ACT7* introduces the possibility of a new role for PKL and raises the prospect that PKL may co-localize with an epigenetic modification associated with actively transcribed genes such as H3K4me3. Using ZipChIP to compare both PKL-cMYC and H3K4me3 localization at *ACT7* and a heterochromatic locus, *MULE*, we were able to quickly screen through multiple regions to determine if there is any correlation between PKL localization and H3K4me3 enrichment (Figure 4A). In agreement with published genome-wide analyses, H3K4me3 was observed to be enriched at the 5′-ORF of *ACT7*, and no H3K4me3 enrichment was observed at the heterochromatic gene *MULE* (Figure 4B). We observed that although PKL preferentially associated with *ACT7* relative to *MULE*, as reported previously, the association of PKL at *ACT7* was relatively constant in each region examined and did not correlate with H3K4me3 enrichment (compare Figure 4B and C). Therefore, using the ZipChIP method, the localization of both H3K4me3 and PKL could be quickly scanned across genes to show that even though PKL preferentially interacts with chromatin at actively transcribed genes, there is no correlation between PKL interaction and the enrichment of H3K4 trimethylation.

## DISCUSSION

In this study, we describe a new ChIP method that we term ZipChIP, which greatly reduces the time and cost of performing ChIP analysis. The ZipChIP method not only decreases the number of reagents and steps compared to other ChIP methods, but also allows for on-bead qRT-PCR analysis through optimization of the wash buffers that are used. More importantly, the use of ZipChIP can improve sensitivity and enable detection of chromatin-associated proteins and histone post-translational modifications that were previously difficult or nearly impossible to detect.

Only two papers have shown ChIP data for Jhd2 and four papers for Rph1 (35–39). It has been reported that Jhd2 and Rph1 can bind 2-fold or less over background in transcriptionally active intragenic regions, which is considerably lower than our results using ZipChIP (Figure 2) (36). In addition, previous ChIP data indicated that Jhd2 had a potential 5′-ORF enrichment while Rph1 associated evenly across the entire ORF (35,36). In contrast, ZipChIP showed that Jhd2 was evenly detected across the genes analyzed. Interestingly, Rph1 was enriched in the gene body of *PYK1*, while a potential 5′-ORF enrichment was observed for Rph1 at *PMA1*, suggesting two different patterns for Rph1 localization. Furthermore, a 3.3–5.3-fold enrichment of Jhd2 was observed at the actively transcribed genes analyzed compared to the origin of replication, *ARS504*. With this selective enrichment of Jhd2 over *ARS504* and Rph1 over background, the functional role of Jhd2 and Rph1 can now be explored using ZipChIP. For example, by using ZipChIP, it is now possible to elucidate how chromatin-associated proteins, like Jhd2 and Rph1, interact with chromatin and targeted to specific loci. Both demethylases contain PHD fingers that are known to interact with proteins and, interestingly, modified lysine residues (40). With the increased sensitivity of ZipChIP further studies can be performed to determine if these PHD fingers or other domains are involved in nucleosomal binding.

The characterization of the enrichment pattern of various histone PTMs by ChIP has played a key role in understanding their cellular function. One modification that has not been fully characterized, especially in yeast, is H3K4 monomethylation. H3K4 monomethylation has been found to play a complex role dependent on the location of the modification. It has been suggested to play a role at enhancers in *Drosophila* and in mammalian cells to help in the regulation of inactive enhancers to the transition to an active state (41,42). Furthermore, studies have indicated that H3K4 monomethylation at promoters can act to either activate or repress expression (43). Given the dramatically different roles associated with H3K4 monomethylation, further understanding this mark and its contribution to gene expression is important. ChIP-chip of H3K4 monomethylation in yeast previously indicated that H3K4 monomethylation is enriched at the 3′-ORF of actively transcribed genes (8). In contrast, ZipChIP revealed that only half of the actively transcribed genes examined had 3′-ORF enrichment. These data indicate that there are at least two distinct patterns of H3K4 monomethylation on actively transcribed genes, which further illustrates the complexity of H3K4 monomethylation and the roles it may play in transcription. Interestingly, the shorter genes show an 3′-ORF enrichment of H3K4 monomethylation suggesting that gene length could have an effect on the pattern of H3K4 monomethylation.

Several studies have reported that deletion of the E3 ubiqutin ligase, Bre1, or the E2 conjugating enzyme, Rad6, leads to a loss of global H3K4 di- and trimethylation (21,27–32). Interestingly, H2B monoubiquitination is not necessary for all H3K4 monomethylation, which is globally reduced but still present (Supplementary Figure S4B) (21,28,29,32). Using ZipChIP we were able to show that 3′-ORF enrichment of H3K4 monomethylation was lost when either *RAD6* or *BRE1* was deleted. This indicates that H2B monoubiquitination is necessary for H3K4 monomethylation 3′-ORF enrichment in a subset of actively transcribed genes and that the global decrease in H3K4 monomethylation observed in *rad6Δ* and *bre1Δ* strains (Supplementary Figure S4B) may be caused by the loss of this enrichment pattern. Further studies using ZipChIP can now begin to elucidate how H3K4 monomethylation is deposited on chromatin and if different types of enrichment patterns across ORFs exist in yeast and other genomes.

Through the study of H3K4 monomethylation we show that ZipChIP can be used to study the cross-talk between PTMs, but ZipChIP can also be used to quickly test the association between a PTM and a chromatin-associated protein. Studies have found that H3K4 methylation serves as a docking sight for other chromatin-modifying proteins or complexes, such as histone acetyltransferase complexes, histone deacetylase complexes and chromatin remodelers (33,44–48). H3K4 methylation and the proteins shown to associate with it are mostly associated with active transcription. However, a recent publication has shown that the *A. thaliana* chromatin remodeler PKL, which is associated with the establishment of the repressive H3K27 trimethylation mark, is also found to be located at the promoter of genes that are actively transcribed. PKL contains a PHD finger and two chromodomains, which are domains that have been shown to bind to lysine methylation, including H3K4 trimethylation, leading to the possibility that PKL may be associated with H3K4 trimethylation (44–48). Our results show that enrichment of PKL does not correlate with the enrichment of H3K4 trimethylation at the promoter region of *ACT7*. This observation reveals that another feature is likely to be responsible for association of PKL with actively transcribed genes. Whether the same feature contributes to recruitment of PKL at genes enriched for H3K27me3 is of considerable interest. Our data in *Arabidopsis* not only demonstrate that ZipChIP can be used to determine if there is correlation between a PTM and a chromatin-modifying enzyme, they also demonstrate that ZipChIP is a robust approach for organisms with a more complex genome such as zebrafish, *Drosophila* and mammalian cells. As a result, ZipChIP will be of great utility for a wide range of laboratories to assist in the study chromatin and DNA-templated processes.

## SUPPLEMENTARY DATA

Supplementary Data are available at NAR Online.

SUPPLEMENTARY DATA
