# Supplementary material for: Immediate chromatin immunoprecipitation and on-bead quantitative PCR analysis: a versatile and rapid ChIP procedure
Source: Nucleic Acids Res. 2014 Dec 24;43(6):e38. doi: 10.1093/nar/gku1347 (PMC4381045; doi:10.1093/nar/gku1347)
Supplement: SUPPLEMENTARY DATA [file supp_43_6_e38__index.html]

Immediate chromatin immunoprecipitation and on-bead quantitative PCR analysis: a versatile and rapid ChIP procedure — SUPPLEMENTARY DATA 

# Immediate chromatin immunoprecipitation and on-bead quantitative PCR analysis: a versatile and rapid ChIP procedure

## SUPPLEMENTARY DATA

**Files in this Data Supplement:**

- SUPPLEMENTARY DATA
