# Supplementary material for: Immediate chromatin immunoprecipitation and on-bead quantitative PCR analysis: a versatile and rapid ChIP procedure
Source: Nucleic Acids Res. 2014 Dec 24;43(6):e38. doi: 10.1093/nar/gku1347 (PMC4381045; doi:10.1093/nar/gku1347)

Supplementary Information

Supplementary Table S1. Yeast strains used for ZipChIP and standard ChIP procedures

Yeast Strain	Genotype	Reference
BY4741	*MATα his3Δ leu2Δ0 LYS2 met15Δ0 ura3Δ0*	Open Biosystems
SDBY1210	BY4741: *MATα his3Δ leu2Δ0 LYS2 met15Δ0 ura3Δ0 set1Δ::HygMX*	(1)
SDBY1107	BY4741: *MATα his3Δ leu2Δ0 LYS2 met15Δ0 ura3Δ0 JHD2 3xFLAG::KanMX*	(2)
SDBY1319	BY4741: MAT*α his3Δ leu2Δ0 LYS2 met15Δ0 ura3Δ0 RPH1 3xFLAG::KanMX*	This study
*rad6*Δ	BY4741: *MATα his3Δ leu2Δ0 LYS2 met15Δ0 ura3Δ0 rad6Δ::KanMX*	Open Biosystems
*bre1*Δ	BY4741: *MATα his3Δ leu2Δ0 LYS2 met15Δ0 ura3Δ0 bre1Δ::KanMX*	Open Biosystems

Supplementary Table S2. Probe sets for ZipChIP and standard ChIP analysis in *S. cerevisiae*

Primer name	Sequence 5'-3'
PYK1 promoter	/56-FAM/CGAATATCG/ZEN/TTTTGATGGCGAGCCTTT/3IABkFQ/<br>AGAGGTTCTTGGAAATGAAAAGTTAC<br>CCTTCCCATATGATGCTAGGTAC
PYK1 5'	/56-FAM/AGAATTGTA/ZEN/CCCAGGTAGACCATTGGC/3IABkFQ/<br>TTCTGATTTCTGGACCCTTGG<br>CATTGACAACGCCAGAAGTC
PYK1 3'	/56-FAM/CTCTCACTT/ZEN/GTACAGAGGTGTCTTGGGA/3IABkFQ/<br>TCAGAGACAGGTTCCTTTTCG<br>TTACCAGATGCCCAAGAGC
PMA1 promoter	/56-FAM/CCGCTTATG/ZEN/CTCCCCTCCATTAGTT/3IABkFQ/<br>TGACGAAACGTGGTCGATG<br>AAATTAGATGTTAGACGATAATGATAGGAC
PMA1 5'	/56-FAM/TTGCCGCCG/ZEN/GTGAAGCTAGA/3IABkFQ/<br>GACAGTGATAACGATGGTCCAG<br>ACCGTAAGATGGGTCAGTTTG
PMA1 3'	/56-FAM/CGTGTCTGG/ZEN/ATCTGGTCTATCGGTATCT/3IABkFQ/<br>ACTGGACTGATATTGTTACTGTCG<br>CATCAATCTGTCAAAGGCTTCAG
MDH2 promoter	/56-FAM/AAAGTCAAA/ZEN/TCGGACCTTCCCAACCT/3IABkFQ/<br>TTTGTTTAGTGGCATTTTGTGTTG<br>CCTTAGATACCTTCCGTAGTTCC
MDH2 5'	/56-FAM/TGGTATCGG/ZEN/GCAGTCGTTATCGC/3IABkFQ/<br>CTGGTATTGCAACTGAGCTTTC<br>ATTGCCATTTTAGGTGCTGC
MDH2 3'	/56-FAM/ATTATTGCC/ZEN/TCTGGTGGACGGTGC/3IABkFQ/<br>AACTTCCCCATTGCTCCTG<br>GTTGCGTTCCATGTCGTTC
HMG1 promoter	/56-FAM/AGTGCGAAG/ZEN/AAAACGTAGTGCGAGT/3IABkFQ/<br>GAAGAGTTGGAAGACCTTCAGC<br>AGATCCTATAGCTAGTACGGACC

HMG1 5’	/56-FAM/TGAATGACG/ZEN/GATAGATAAGCGAATGCGG/3IABkFQ/<br>CGGCGAAACGACCAATTC<br>GTGTTGGAGTCTTTATTTGGAGC
HMG1 3’	/56-FAM/AGGTTTTGT/ZEN/TGGTTCAGCAGGTTTCC/3IABkFQ/<br>ACGATTTATATCAGTGGCGTCC<br>CGGCCATTTGGTTCAAAGTC
ERG11 promoter	/56-FAM/TCGTTAACT/ZEN/CGTGGAGATGCACAATAGG/3IABkFQ/<br>GCAGGAGACATCGATTTTATGC<br>GAGGCTTTTCGAATACATGCG
ERG11 5’	/56-FAM/TGGGCCAAT/ZEN/GGTAAAGCCAAGAAATG/3IABkFQ/<br>TTGGAGAGGCATTGGAATACG<br>TCCTTTCTCAAAGAATATAGTAATTGCC
ERG11 3’	/56-FAM/ACCGTTCCA/ZEN/CCTCCTGACTTTACATCT/3IABkFQ/<br>AAATGGCATTACCCAGAGGG<br>TTTGTTCTGGATTTCTCTTTTCCC
HMG2 promoter	/56-FAM/CCCTTTGGT/ZEN/ACTCCACCTCTTCAAGATG/3IABkFQ/<br>CACCTCAAGAACCTACCACAC<br>AGTGTCCTTACTATAACTTGCTCG
HMG2 5’	/56-FAM/CTCAGCTCT/ZEN/AAAGAAGCCGCCGAT/3IABkFQ/<br>GGTCTCCTGTGTCTGATACATG<br>TCAAGGGAAGGCAAAGTCG
HMG2 3’	/56-FAM/CCGGTCACC/ZEN/TGGTACAAAGCCAT/3IABkFQ/<br>GTGAACTGTCTCTGTGCTCC<br>GCCCTTTGTTACTTGGTTGTG
YFR057W upstream intergenic	/56-FAM/TGCCATGTA/ZEN/TGCAACTGCTCTACCA/3IABkFQ/<br>ACACCCGTCTTTGATGCTAG<br>ATGTTCTCACGCTCTGTTCTAG
YFR057W promoter	/56-FAM/ACAAGGGAA/ZEN/CAATGAGCAGAGGGAAA/3IABkFQ/<br>CAAACAAGTAGGAATGCGAAAGG<br>AAGTGCTAAAGGAATCCCCAG
YFR057W 5’	/56-FAM/TGCTCGGCC/ZEN/AAGTCAAGCG/3IABkFQ/<br>GCTTGGCGGTGTCTTTAATG<br>TGATATTTGGACCTACTAGTGTCTATAG
YFR057W 3’	/56-FAM/TGTCAAAGG/ZEN/AATTTTTTGCGCCTAGTGC/3IABkFQ/<br>AAAGGATTTTAGCAACGACTTCG<br>TTGTCGAATCTTCCAGTAAATTTCTC
YFR057W 3’-UTR	/56-FAM/ACTTTCTGG/ZEN/AATAGCGTTCGGAATGTGT/3IABkFQ/<br>CACTCGTTAGGATCACGTTCG<br>TGTCGGAGAGTTAACAAGCG
ARS504	/56-FAM/TTGGTCTTC/ZEN/CCTACGCATTTCCCC/3IABkFQ/<br>GCCGTTTCAATCTATCGTCATG<br>ATCCAGCTGACTCATTTCCTG

Supplementary Table S3. Primers for ZipChIP analysis in *A. thaliana*

Gene	Region	Forward primer sequence 5’-3’	Reverse primer sequence 5’-3’
ACT7	1	TTTCGGCCCGTTTTATCGT	GAGCCACCATGCTTCTCATT
ACT7	2	TGGGGGTTGCTTGTTATGTG	AACAGCGAGAGATCGACAGA
ACT7	3	AATACAGTGTCTGATCGGAGGAT	GCGGAATTGGGATTTTTACCT
ACT7	4	GAATAGTCCCTCGGGGTTGA	TCGTGTTGGAATCAGTTGATGA
MULE	1	CGAAGCGTTTGATCGGGTAT	TTCGACTGTTTACCAACTGGA

MULE	2	CTTGGAACTTCCCGAGAGCTT	CACCACTTCACAAACCCACCTA
MULE	3	CCCATGATTGACAGTCGTTGA	AGGGCTATGAGTTGCCAAGA
MULE	4	ACACCTTCGAGACTTCCCTT	TGTACAGCGGCGTTAAAACA

Supplementary Table S4. ChIP analysis of H3K4me3 comparing ZipChIP to standard ChIP

Procedure	Gene location	Fold change relative to *set1Δ*	P value
ZipChIP	*PYK1* promoter	44.29 +/- 15.94	0.053
ZipChIP	*PYK1* 5’	216.79 +/- 33.37	0.003
ZipChIP	*PYK1* 3’	23.88 +/- 5.72	0.016
Standard	*PYK1* promoter	47.83 +/- 14.28	0.031
Standard	*PYK1* 5’	161.06 +/- 37.43	0.013
Standard	*PYK1* 3’	26.30 +/- 6.41	0.017
ZipChIP	*PMA1* promoter	50.90 +/- 14.12	0.024
ZipChIP	*PMA1* 5’	107.70 +/- 28.40	0.020
ZipChIP	*PMA1* 3’	15.17 +/- 5.56	0.063
Standard	*PMA1* promoter	49.25 +/- 11.84	0.015
Standard	*PMA1* 5’	91.88 +/- 17.18	0.006
Standard	*PMA1* 3’	10.37 +/- 1.80	0.007
ZipChIP	*YFR057W* promoter	7.34 +/- 2.58	0.070
ZipChIP	*YFR057W* 5’	5.72 +/- 1.65	0.046
ZipChIP	*YFR057W* 3’	7.62 +/- 3.74	0.151
Standard	*YF057W* promoter	6.26 +/- 1.57	0.028
Standard	*YFR057W* 5’	5.79 +/- 1.42	0.028
Standard	*YFR057W* 3’	6.94 +/- 1.55	0.019

Supplementary Table S5. ChIP analysis of H3K4me3 in a *set1*Δ strain comparing ZipChIP to standard ChIP

Procedure	Gene location	Fold change relative to individual gene promoter
ZipChIP	*PYK1* promoter	
ZipChIP	*PYK1* 5’	1.51 +/- 0.24
ZipChIP	*PYK1* 3’	1.47 +/- 0.95
Standard	*PYK1* promoter	
Standard	*PYK1* 5’	1.64 +/- 0.70
Standard	*PYK1* 3’	1.16 +/- 0.09
ZipChIP	*PMA1* promoter	
ZipChIP	*PMA1* 5’	2.08 +/- 0.32
ZipChIP	*PMA1* 3’	1.90 +/- 0.58
Standard	*PMA1* promoter	
Standard	*PMA1* 5’	1.41 +/- 0.51
Standard	*PMA1* 3’	-1.41 +/- 0.49
ZipChIP	*YFR057W* promoter	
ZipChIP	*YFR057W* 5’	-1.06 +/- 0.09
ZipChIP	*YFR057W* 3’	1.03 +/- 0.22
Standard	*YF057W* promoter	
Standard	*YFR057W* 5’	-1.04 +/- 0.13
Standard	*YFR057W* 3’	-1.05 +/- 0.20

Supplementary Table S6. ChIP analysis of H3K4me3 in a WT strain comparing ZipChIP to standard ChIP

Procedure	Gene location	Fold change relative to individual gene promoter
ZipChIP	*PYK1* promoter	
ZipChIP	*PYK1* 5’	9.39 +/- 2.14
ZipChIP	*PYK1* 3’	-1.21 +/- 0.06
Standard	*PYK1* promoter	
Standard	*PYK1* 5’	5.04 +/- 0.56
Standard	*PYK1* 3’	-1.64 +/- 0.06
ZipChIP	*PMA1* promoter	
ZipChIP	*PMA1* 5’	4.46 +/- 0.65
ZipChIP	*PMA1* 3’	-2.24 +/- 0.02
Standard	*PMA1* promoter	
Standard	*PMA1* 5’	2.25 +/- 0.50
Standard	*PMA1* 3’	-6.92 +/- 0.10
ZipChIP	*YFR057W* promoter	
ZipChIP	*YFR057W* 5’	-1.33 +/- 0.02
ZipChIP	*YFR057W* 3’	-1.06 +/- 0.27
Standard	*YF057W* promoter	
Standard	*YFR057W* 5’	-1.12 +/- 0.11
Standard	*YFR057W* 3’	-1.55 +/- 0.26

Supplementary Table S7. ChIP analysis of H3K4me3 comparing ZipChIP to standard ChIP

Procedure	Gene location	Fold change relative to YFR057W promoter
ZipChIP	*PYK1* promoter	6.58 +/- 1.10
ZipChIP	*PYK1* 5’	57.06 +/- 4.13
ZipChIP	*PYK1* 3’	5.31 +/- 0.50
Standard	*PYK1* promoter	8.66 +/- 0.64
Standard	*PYK1* 5’	43.04 +/- 0.83
Standard	*PYK1* 3’	5.36 +/- 0.85
ZipChIP	*PMA1* promoter	5.89 +/- 0.52
ZipChIP	*PMA1* 5’	26.69 +/- 5.83
ZipChIP	*PMA1* 3’	2.62 +/- 0.28
Standard	*PMA1* promoter	8.19 +/- 1.30
Standard	*PMA1* 5’	17.23 +/- 1.87
Standard	*PMA1* 3’	0.94 +/- 0.48

Supplementary Table S8. ChIP analysis of Jhd2-3xFLAG comparing ZipChIP to standard ChIP

Procedure	Gene location	Fold change relative to WT	P value
ZipChIP	*PYK1* promoter	11.37 +/- 2.51	0.015
ZipChIP	*PYK1* 5’	11.45 +/- 0.33	6.21×10^{-6}
ZipChIP	*PYK1* 3’	13.50 +/- 3.25	0.018
Standard	*PYK1* promoter	1.17 +/- 0.15	0.330
Standard	*PYK1* 5’	-1.03 +/- 0.18	0.874
Standard	*PYK1* 3’	-1.02 +/- 0.11	0.851

ZipChIP	*PMA1* promoter	10.33 +/- 4.85	0.127
ZipChIP	*PMA1* 5’	8.40 +/- 1.03	0.002
ZipChIP	*PMA1* 3’	9.68 +/- 0.62	0.0002
Standard	*PMA1* promoter	1.05 +/- 0.09	0.651
Standard	*PMA1* 5’	1.03 +/- 0.19	0.889
Standard	*PMA1* 3’	-1.03 +/- 0.14	0.809
ZipChIP	*ARS504*	2.54 +/- 0.43	0.024

Supplementary Table S9. Fold difference of Jhd2-3xFLAG levels at *PYK1* and *PMA1* compared to *ARS504*

Gene location	Fold change relative to *ARS504* locus
ARS504	1
PYK1 promoter	4.48
PYK1 5’	4.51
PYK1 3’	5.32
ARS504	1
PMA1 promoter	4.07
PMA1 5’	3.31
PMA1 3’	3.82

Supplementary Table S10. ChIP analysis of Rph1-3xFLAG comparing ZipChIP to standard ChIP

Procedure	Gene location	Fold change relative to WT	P value
ZipChIP	*PYK1* promoter	9.26 +/- 1.52	0.006
ZipChIP	*PYK1* 5’	16.96 +/- 2.27	0.002
ZipChIP	*PYK1* 3’	19.55 +/- 2.80	0.003
Standard	*PYK1* promoter	1.15 +/- 0.24	0.566
Standard	*PYK1* 5’	1.35 +/- 0.24	0.219
Standard	*PYK1* 3’	1.32 +/- 0.29	0.335
ZipChIP	*PMA1* promoter	15.47 +/- 3.72	0.018
ZipChIP	*PMA1* 5’	24.92 +/- 3.00	0.001
ZipChIP	*PMA1* 3’	20.53 +/- 1.77	0.0004
Standard	*PMA1* promoter	1.30 +/- 0.38	0.476
Standard	*PMA1* 5’	1.28 +/- 0.25	0.316
Standard	*PMA1* 3’	1.30 +/- 0.13	0.079
ZipChIP	*ARS504*	17.41 +/- 5.88	0.049

Supplementary Table S11. ZipChIP analysis of Sir2

Gene location	Region	Fold change relative to No Ab
YFR057W upstream intergenic	1	1.45 +/- 0.17
YFR057W promoter	2	1.26 +/- 0.73
YFR057W 5’	3	4.04 +/- 0.28
YFR057W 3’	4	6.11 +/- 1.85
YFR057W 3’ UTR	5	7.44 +/- 0.82
PYK1 3’	NA	1.81 +/- 0.36
PMA1 3’	NA	1.59 +/- 0.18

Supplementary Table S12. ZipChIP analysis of H3K4me1

Gene location	Fold change relative to *set1Δ*	P value
PYK1 promoter	4.76 +/- 1.36	0.051
PYK1 5'	4.87 +/- 0.90	0.012
PYK1 3'	24.73 +/- 5.55	0.013
MDH2 promoter	3.43 +/- 0.18	0.0002
MDH2 5'	8.91 +/- 0.75	0.0005
MDH2 3'	31.66 +/- 4.72	0.003
PMA1 promoter	5.12 +/- 1.48	0.050
PMA1 5'	8.60 +/- 1.21	0.003
PMA1 3'	5.31 +/- 0.32	0.003
HMG1 promoter	8.99 +/- 2.43	0.030
HMG1 5'	6.56 +/- 1.63	0.027
HMG1 3'	13.07 +/- 2.66	0.011
ERG11 promoter	9.48 +/- 1.13	0.0003
ERG11 5'	9.16 +/- 1.80	0.004
ERG11 3'	24.33 +/- 6.65	0.013
HMG2 promoter	8.79 +/- 1.77	0.005
HMG2 5'	18.67 +/- 4.00	0.005
HMG2 3'	14.61 +/- 3.39	0.007

Supplementary Table S13. ZipChIP analysis of H3K4me1 in a *rad6*Δ and *bre1*Δ

*rad6*Δ		*bre1*Δ	
Gene location	Fold change relative to *set1*Δ	Gene location	Fold change relative to *set1*Δ
PYK1 promoter	4.23 +/- 2.39	*PYK1* promoter	2.61 +/- 1.08
PYK1 5'	4.11 +/- 0.27	*PYK1* 5'	5.11 +/- 0.78
PYK1 3'	1.47 +/- 0.21	*PYK1* 3'	1.89 +/- 0.29
MDH2 promoter	2.34 +/- 0.61	*MDH2* promoter	2.21 +/- 0.66
MDH2 5'	3.47 +/- 0.34	*MDH2* 5'	6.02 +/- 2.65
MDH2 3'	4.56 +/- 2.76	*MDH2* 3'	5.04 +/- 2.85
PMA1 promoter	2.33 +/- 0.28	*PMA1* promoter	1.45 +/- 0.34
PMA1 5'	2.63 +/- 0.69	*PMA1* 5'	3.55 +/- 1.06
PMA1 3'	1.99 +/- 0.98	*PMA1* 3'	1.39 +/- 1.06
HMG1 promoter	4.14 +/- 1.24	*HMG1* promoter	5.76 +/- 3.37
HMG1 5'	9.14 +/- 1.91	*HMG1* 5'	10.87 +/- 2.88
HMG1 3'	6.33 +/- 2.85	*HMG1* 3'	5.64 +/- 2.45

Supplementary Table S14. ZipChIP analysis of H3K4me3 in *A. thaliana*

Gene	Region	ΔCt
ACT7	1	0.14 +/- 0.01
ACT7	2	0.65 +/- 0.08
ACT7	3	0.16 +/- 0.03
ACT7	4	0.15 +/- 0.01
MULE	1	0.06 +/- 0.01
MULE	2	0.06 +/- 0.01
MULE	3	0.06 +/- 0.01

MULE	4	0.11 +/- 0.03

Supplementary Table S15. ZipChIP analysis of MYC-PKL in *A. thaliana*

Gene	Region	Fold change relative to WT	P value
ACT7	1	4.58 +/- 0.40	0.039
ACT7	2	5.62 +/- 1.79	0.019
ACT7	3	3.65 +/- 0.75	0.050
ACT7	4	8.00 +/- 2.76	0.015
MULE	1	1.83 +/- 0.29	0.051
MULE	2	1.83 +/- 0.51	0.082
MULE	3	1.87 +/- 0.14	0.072
MULE	4	2.48 +/- 0.52	0.087

REFERENCES

1. South,P.F., Fingerman,I.M., Mersman,D.P., Du,H.N. and Briggs,S.D. (2010) A conserved interaction between the SDI domain of Bre2 and the Dpy-30 domain of Sdc1 is required for histone methylation and gene expression. *J. Biol. Chem.*, **285**, 595-607.

2. Mersman,D.P., Du,H.N., Fingerman,I.M., South,P.F. and Briggs,S.D. (2009) Polyubiquitination of the demethylase Jhd2 controls histone methylation and gene expression. *Genes Dev.*, **23**, 951-962.

SUPPLEMENTARY LEGENDS

Supplementary Figure S1. ChIP analysis of H3K4 trimethylation using both ZipChIP and standard ChIP showed similar enrichment of H3K4 trimethylation. PrimeTime qPCR probes were targeted toward the promoter (P), 5'-ORF (5), and 3'-ORF (3) of two actively transcribed genes *PYK1* and *PMA1*, as well as a sub-telomere gene *YFR057W* comparing ZipChIP and standard ChIP methods. ChIP analysis was performed on BY4741 wild-type (WT) strain using antibodies specific for H3K4me3 and histone H3. Input and histone H3 were used for normalization. (**A**-**C**) ChIP analysis of H3K4 trimethylation in a *set1Δ* strain is relative to the H3K4me3 signal in a *set1Δ* strain for the promoter of each individual gene. (**D**-**F**) ChIP analysis of H3K4 trimethylation in a WT strain is relative to the H3K4me3 signal in a WT strain for the promoter of each individual gene. (**G** and **H**) H3K4me3 ChIP analysis in a WT strain is relative to the H3K4me3 signal for the promoter of *YFR057W* in a WT strain. Three biological replicates with three technical repeats were used for all ZipChIP and standard ChIP analysis. The error bars represent the standard error of the mean.

Supplementary Figure S2. Low levels of Jhd2 but not Rph1 were observed at *ARS504*. PrimeTime qPCR probes were targeted toward the *ARS504*, an origin of replication on chromosome V, was used for ZipChIP analysis of BY4741 WT strain and a *JHD2*-3xFLAG strain (**A**) and a *RPH1*-3xFLAG strain (**C**). Three biological replicates with three technical repeats were used for all ZipChIP analysis. The error bars represent the standard error of the mean. (**B**) The fold difference of Jhd2-3xFLAG binding was calculated to compare the levels of Jhd2 at *PYK1* and *PMA1* to Jhd2 levels observed at *ARS504*.

Supplementary Figure S3. ZipChIP can detect enrichment of the histone deacetylase Sir2 at the sub-telomere gene *YFR057W*. (**A**) ChIP analysis using a Sir2 specific antibody was used to analyze Sir2 enrichment over five regions of *YFR057W*. Region 1 was upstream of *YFR057W* in an intergenic region. Region 2 was in the *YFR057W* promoter. Region 3 and 4 were in the 5' and 3'-ORF region of *YFR057W*, respectively. Region 5 was in the 3'-UTR of *YFR057W*. (**B** and **C**) ChIP analysis of Sir2 was used to analyze Sir2 localization at the 3'-ORF of two actively transcribed genes *PYK1* (**B**) and *PMA1* (**C**). Sir2 signal was set relative to a mock ChIP experiment with no antibody conjugated to the magnetic beads (No Ab). Three biological replicates with three technical repeats were used for all ZipChIP analysis. The error bars represent the standard error of the mean.

Supplementary Figure S4. Two distinct patterns of H3K4 monomethylation were found using ZipChIP and global H3K4 methylation levels are affected in *RAD6* and *BRE1* deletion strains. (**A**) PrimeTime qPCR probes were targeted toward the promoter (P), 5'-ORF (5), and 3'-ORF (3) of *ERG11* and *HMG2*. Four biological replicates with three technical repeats were used for all ZipChIP analysis. The error bars represent the standard error of the mean. (**B**) Global H3K4 monomethylation is reduced in *rad6*Δ and *bre1*Δ strains. Immunoblots of whole cell extracts from *set1*Δ, WT, *rad6*Δ, and *bre1*Δ strains were analyzed using methylation specific antibodies targeting H3K4 mono-, di- and trimethylation. A *set1*Δ strain was used as a negative control. H3 antibody was used as a loading control.

Supplementary Figure S1

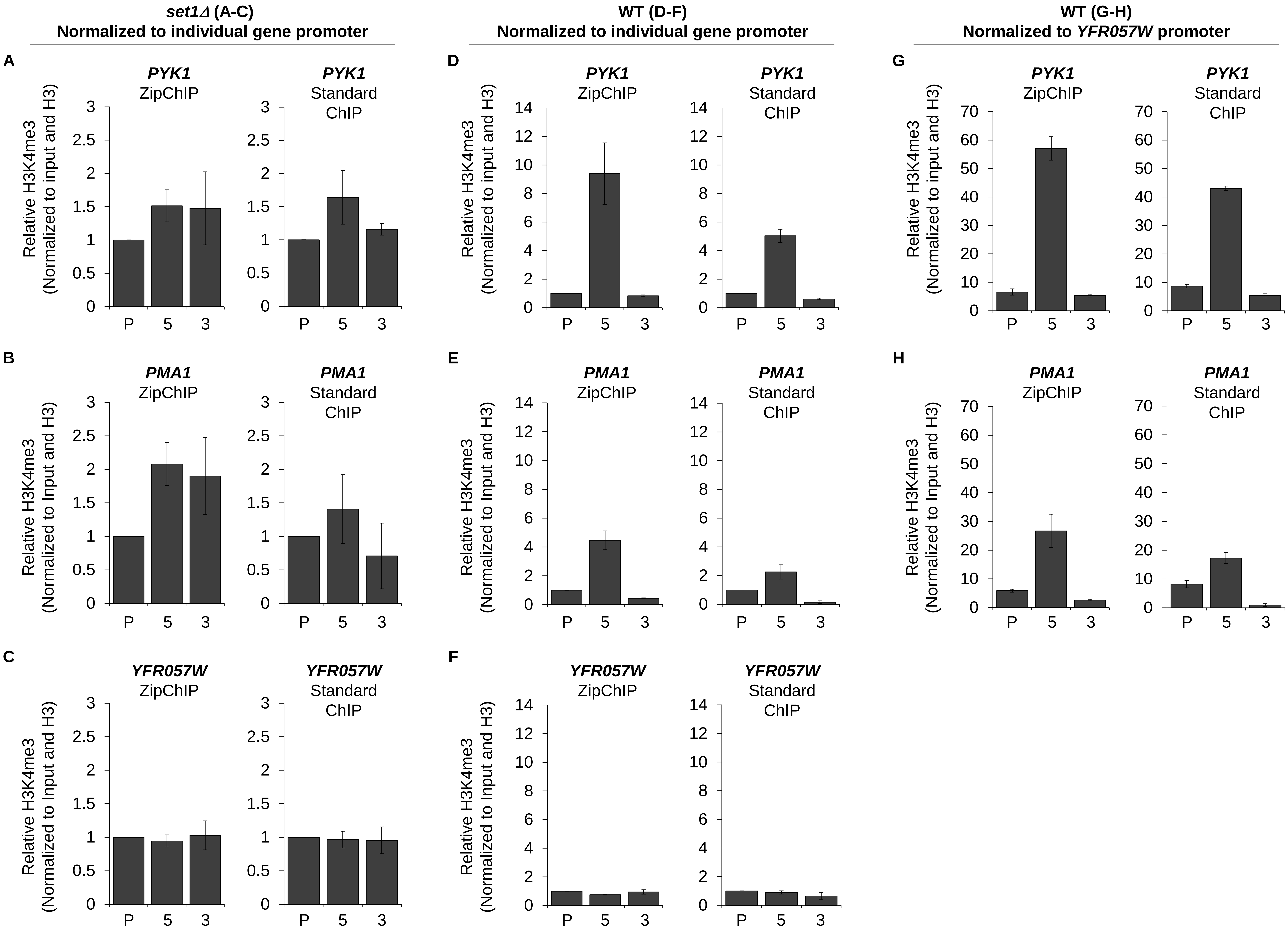

Supplementary Figure S2

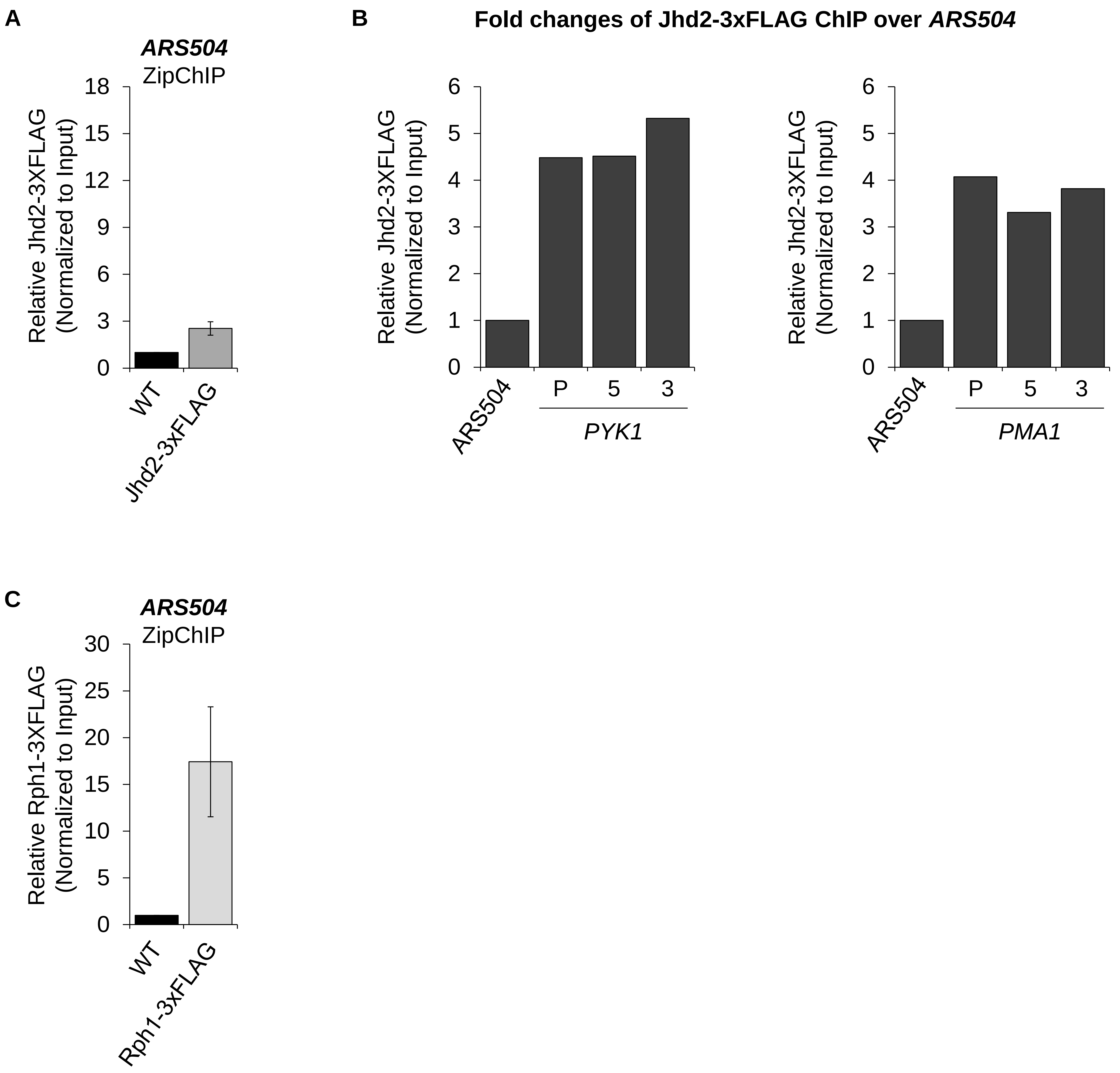

Supplementary Figure S3

A B

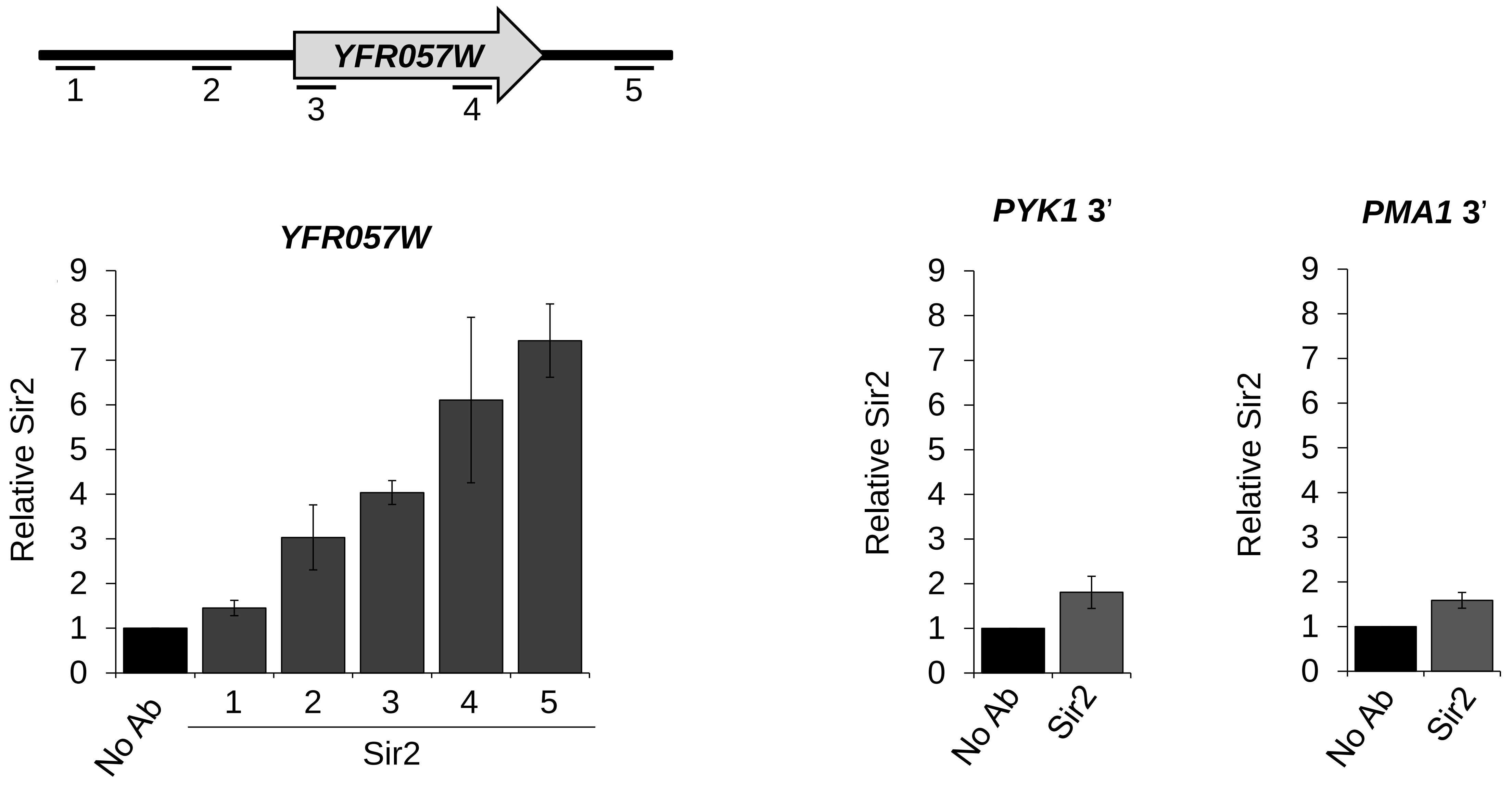

Supplementary Figure S4

A

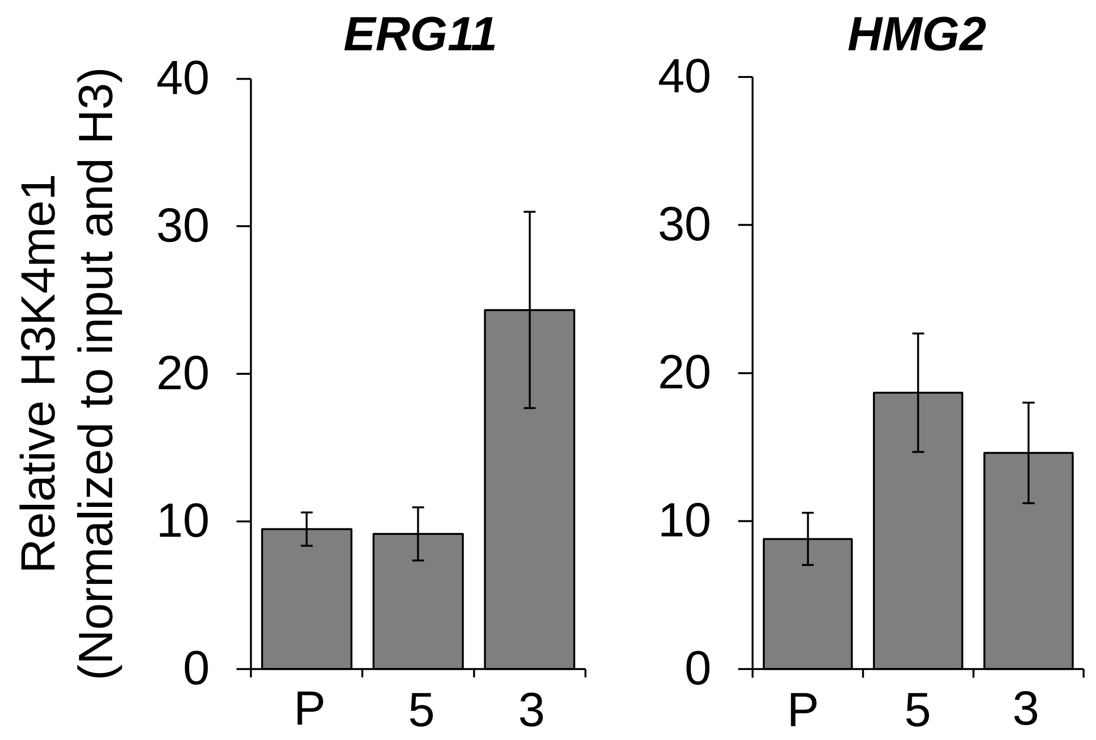

B

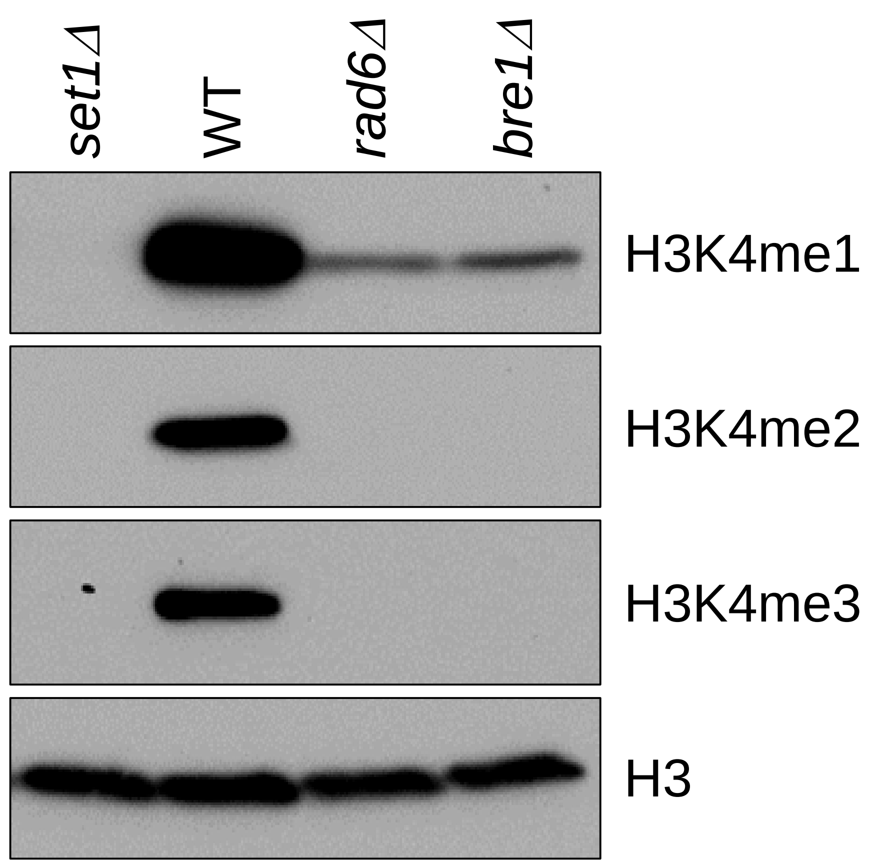

Supplement: SUPPLEMENTARY DATA [file supp_gku1347_ZipChIP-supplementary-Information-and-figures-NAR-revisions01-19-15.pdf]
